# Supplementary figures and images for: Influence of Parameters Used to Prepare Sterile Solutions of Poloxamer 188 on Their Physicochemical Properties
Source: Polymers (Basel). 2024 Dec 29;17(1):62. doi: 10.3390/polym17010062 (PMC11722941; doi:10.3390/polym17010062)

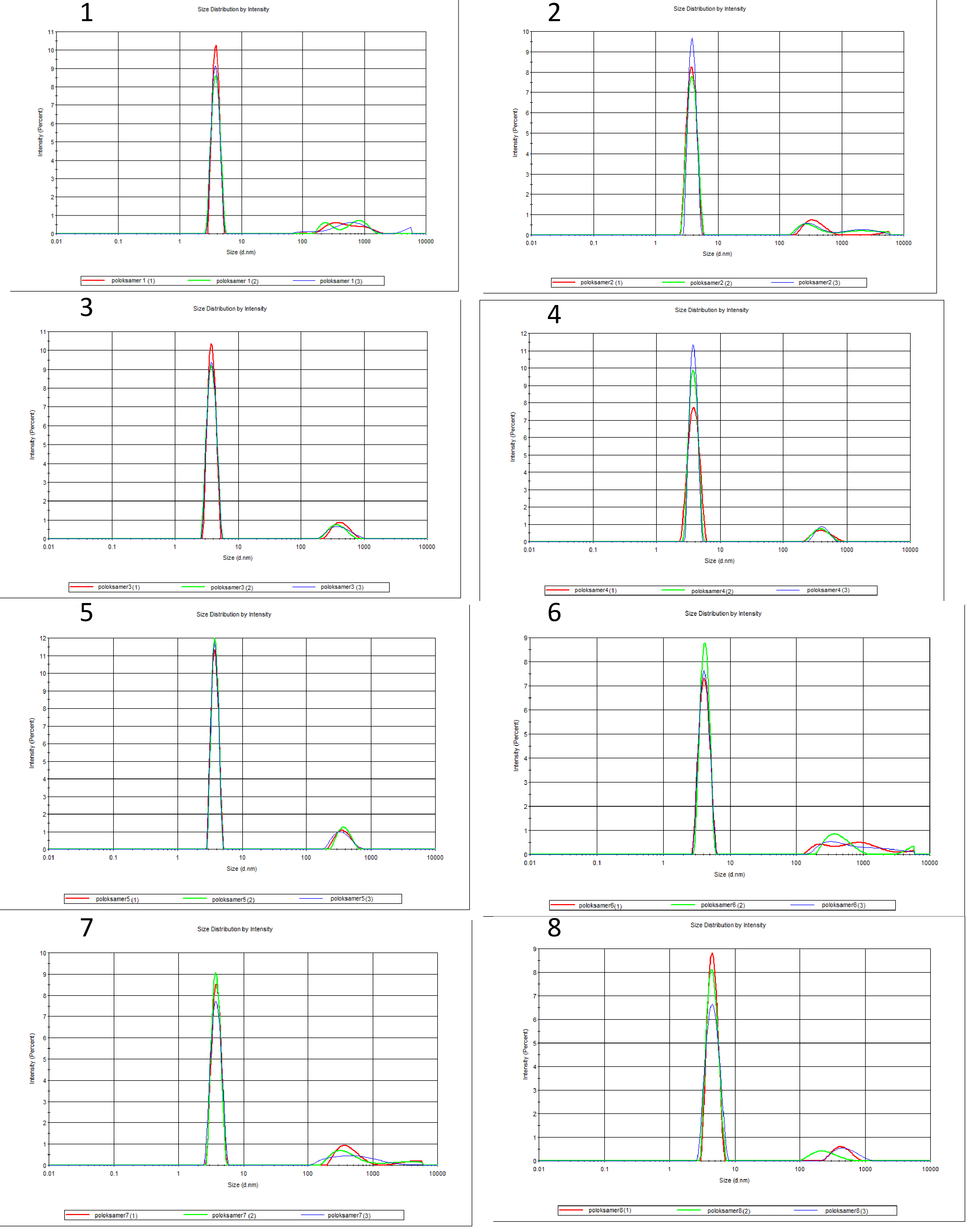

Supplement: Supplementary file 1 [file polymers-17-00062-s001.zip › FigureS1a.png]

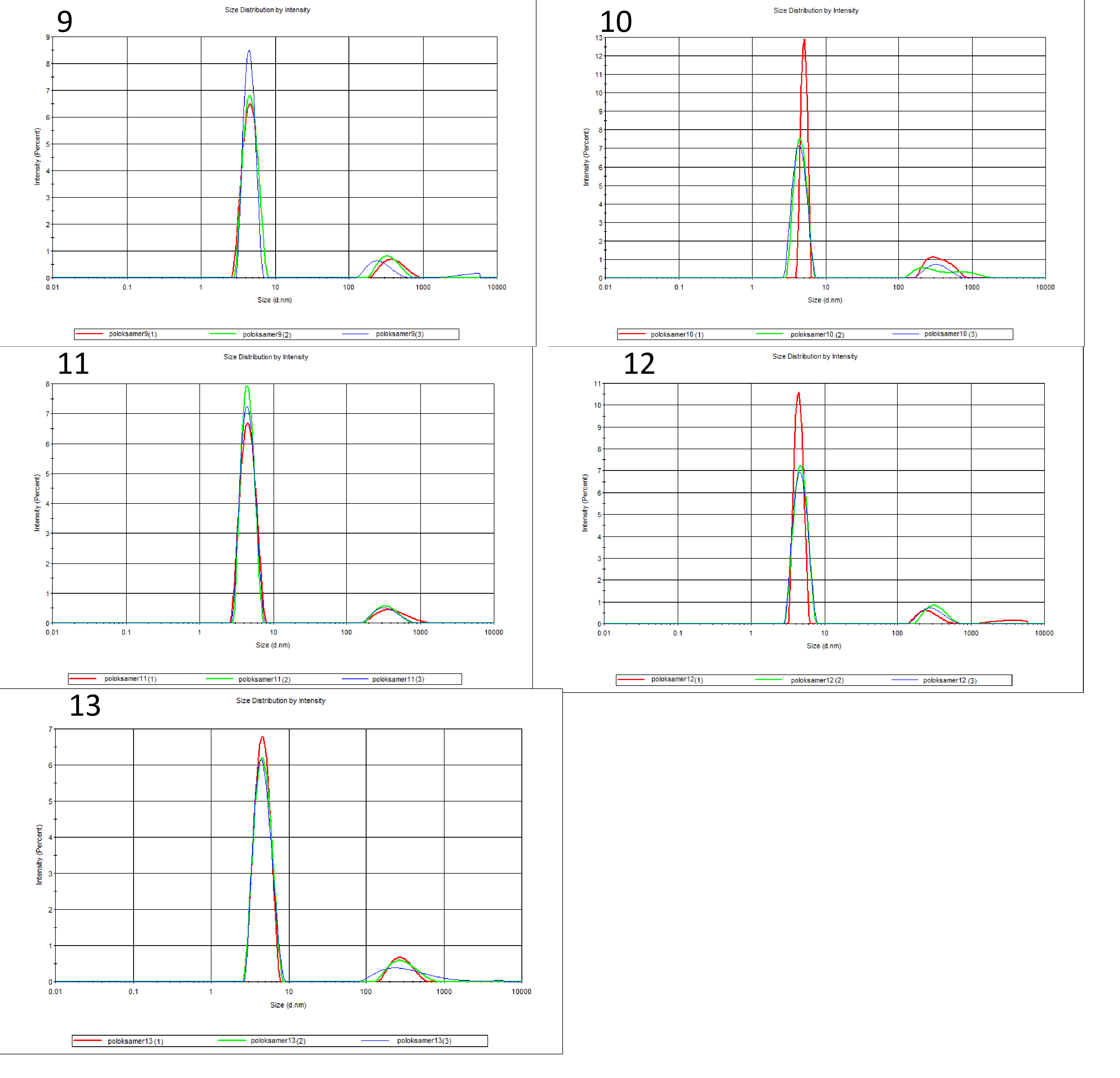

Supplement: Supplementary file 1 [file polymers-17-00062-s001.zip › FigureS1b.png]

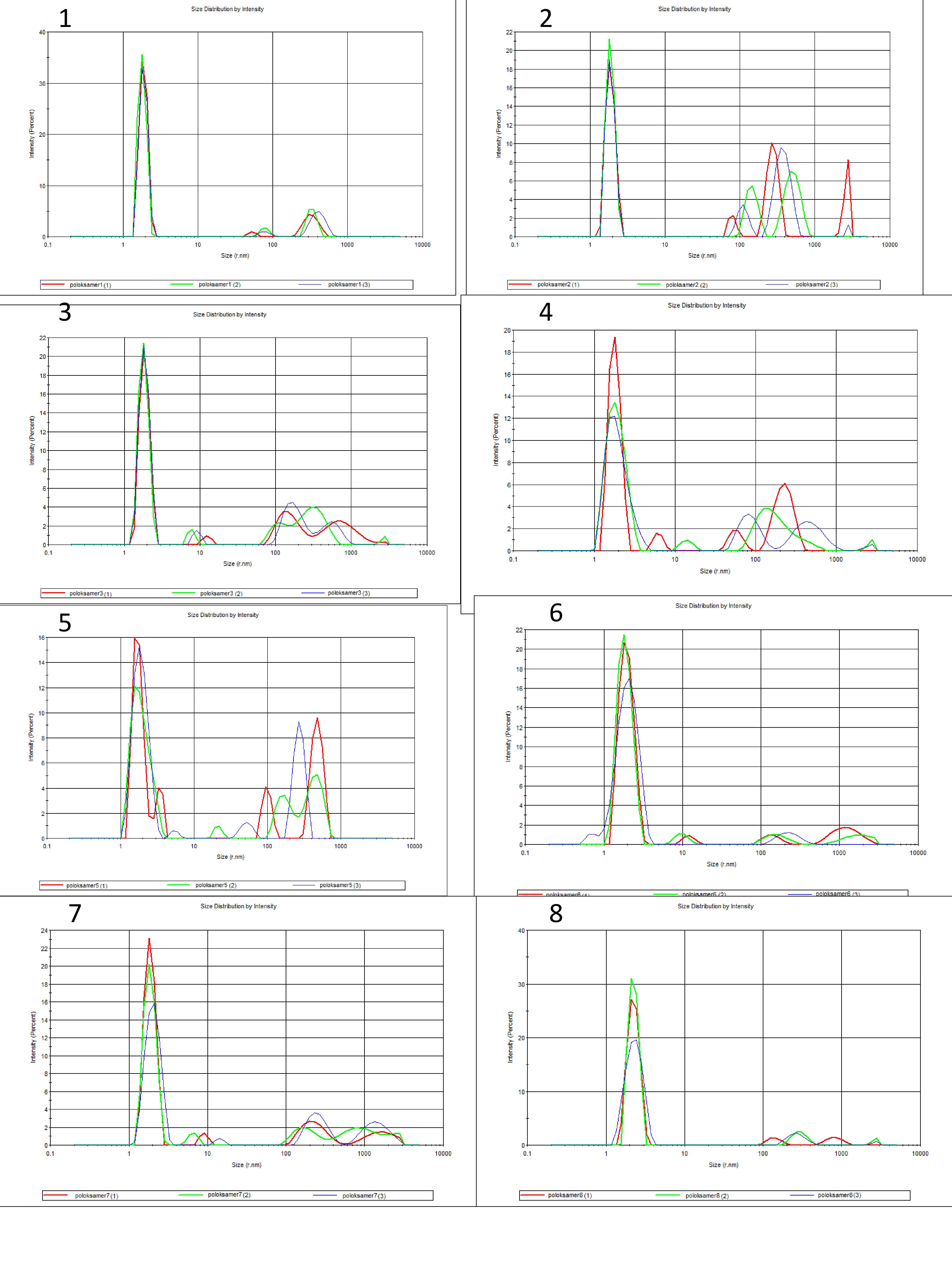

Supplement: Supplementary file 1 [file polymers-17-00062-s001.zip › FigureS2a.png]

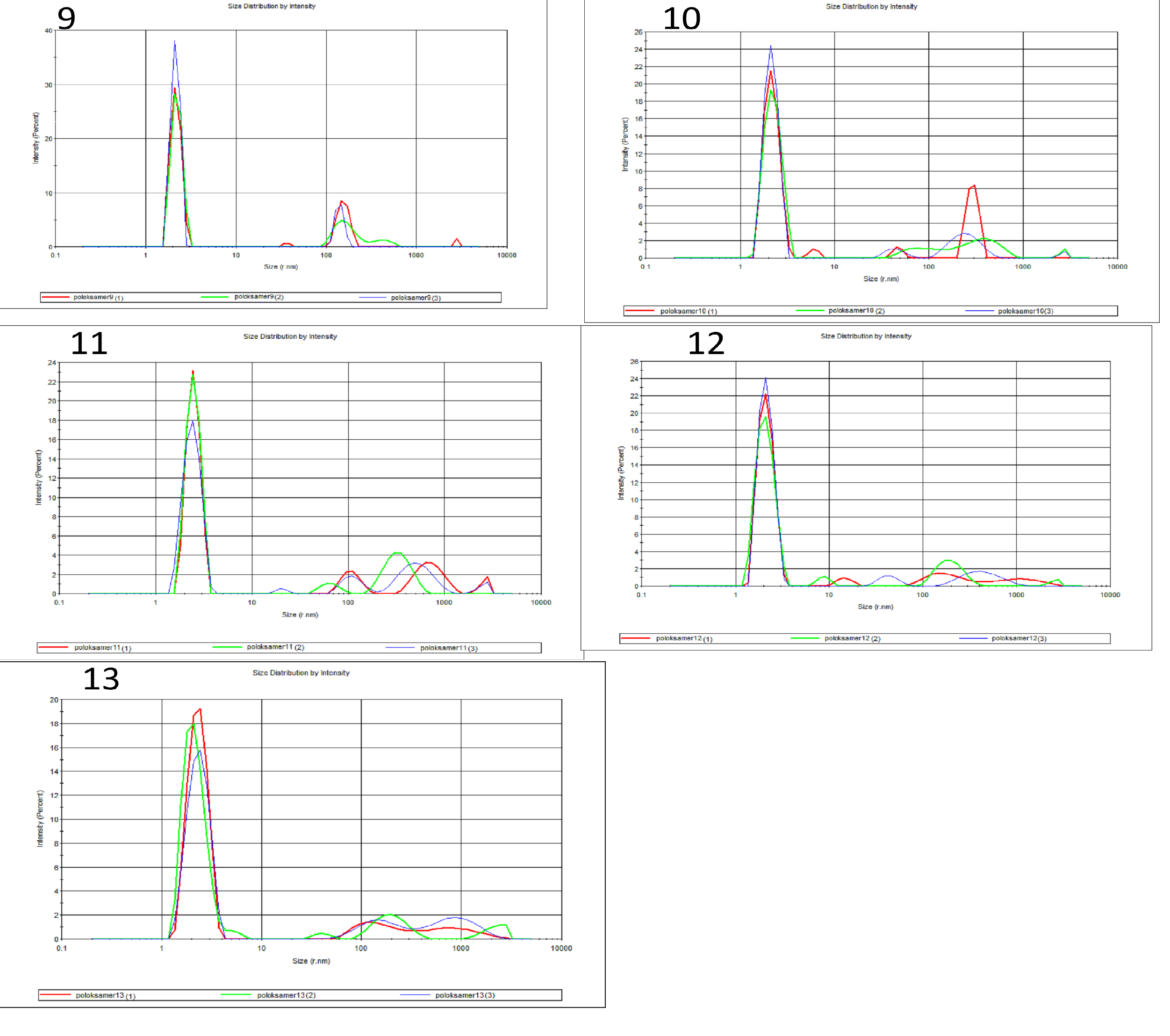

Supplement: Supplementary file 1 [file polymers-17-00062-s001.zip › FigureS2b.png]
